# Exogenous Myo-Inositol Alleviates Salt Stress by Enhancing Antioxidants and Membrane Stability via the Upregulation of Stress Responsive Genes in *Chenopodium quinoa* L.

**DOI:** 10.3390/plants10112416

**Published:** 2021-11-09

**Authors:** Amina A. M. Al-Mushhin, Sameer H. Qari, Marwa A. Fakhr, Ghalia S. H. Alnusairi, Taghreed S. Alnusaire, Ayshah Aysh ALrashidi, Arafat Abdel Hamed Abdel Latef, Omar M. Ali, Amir Abdullah Khan, Mona H. Soliman

**Affiliations:** 1 Department of Biology, College of Science and Humanities in Al-Kharj, Prince Sattam Bin Abdulaziz University, Al-Kharj 11942, Saudi Arabia; a.almushhin@psau.edu.sa; 2 Biology Department, Al-Jumum University College, Umm Al-Qura University, Mecca 21955, Saudi Arabia; shqari@uqu.edu.sa; 3 Botany Department, Faculty of Science, Fayoum University, Fayoum 63514, Egypt; maa29@fayoum.edu.eg; 4 Plant Protection and Bimolecular Diagnosis Department, Arid Lands Cultivation Research Institute, City of Scientific Research and Technological Application (SRTA-city), New Borg El-Arab City 21934, Egypt; 5 Department of Biology, College of Science, Jouf University, Sakaka 2014, Saudi Arabia; gshalnusairi@ju.edu.sa (G.S.H.A.); taghreed0804@hotmail.com (T.S.A.); 6 Department of Biology, Faculty of Science, University of Hail, Hail 81411, Saudi Arabia; ais.alrashydy@uoh.edu.sa; 7 Botany and Microbiology Department, Faculty of Science, South Valley University, Qena 83523, Egypt; moawad76@gmail.com; 8 Department of Chemistry, Turabah University College, Turabah Branch, Taif University, P.O. Box 11099, Taif 21944, Saudi Arabia; om.ali@tu.edu.sa; 9 Department of Plant Biology and Ecology, Nankai University, Tianjin 300071, China; 10 Botany and Microbiology Department, Faculty of Science, Cairo University, Giza 12613, Egypt; hmona@sci.cu.edu.eg; 11 Biology Department, Faculty of Science, Taibah University, Al-Sharm, Yanbu El-Bahr, Yanbu 46429, Saudi Arabia

**Keywords:** ascorbate–glutathione cycle, gene expression, myo-inositol, photosynthetic attributes, salinity stress, physiological mechanisms

## Abstract

Myo-inositol has gained a central position in plants due to its vital role in physiology and biochemistry. This experimental work assessed the effects of salinity stress and foliar application of myo-inositol (MYO) on growth, chlorophyll content, photosynthesis, antioxidant system, osmolyte accumulation, and gene expression in quinoa (*Chenopodium quinoa* L. var. Giza1). Our results show that salinity stress significantly decreased growth parameters such as plant height, fresh and dry weights of shoot and root, leaf area, number of leaves, chlorophyll content, net photosynthesis, stomatal conductance, transpiration, and Fv/Fm, with a more pronounced effect at higher NaCl concentrations. However, the exogenous application of MYO increased the growth and photosynthesis traits and alleviated the stress to a considerable extent. Salinity also significantly reduced the water potential and water use efficiency in plants under saline regime; however, exogenous application of myo-inositol coped with this issue. MYO significantly reduced the accumulation of hydrogen peroxide, superoxide, reduced lipid peroxidation, and electrolyte leakage concomitant with an increase in the membrane stability index. Exogenous application of MYO up-regulated the antioxidant enzymes’ activities and the contents of ascorbate and glutathione, contributing to membrane stability and reduced oxidative damage. The damaging effects of salinity stress on quinoa were further mitigated by increased accumulation of osmolytes such as proline, glycine betaine, free amino acids, and soluble sugars in MYO-treated seedlings. The expression pattern of OSM34, NHX1, SOS1A, SOS1B, BADH, TIP2, NSY, and SDR genes increased significantly due to the application of MYO under both stressed and non-stressed conditions. Our results support the conclusion that exogenous MYO alleviates salt stress by involving antioxidants, enhancing plant growth attributes and membrane stability, and reducing oxidative damage to plants.

## 1. Introduction

Salinity is considered a negative factor that significantly reduces crop plant growth, development, and yield potential [1,2]. Utilization of saline water for irrigating agricultural land has further aggravated the situation, resulting in the conversion of fertile and productive lands into unproductive lands [3], which in turn has led to a severe impact on global food security. According to the FAO, nearly 6% of the world’s land area is saline affected [4]. Salinity stress is the presence of excess salt ions in soil solution, resulting in ionic and osmotic stress [4]. Salinity stress restricts root growth, resulting in limited access to mineral ions, and impedes membrane stability by triggering lipid and protein peroxidation. It also downregulates the functioning of enzymes and triggers chlorophyll damage and photosynthetic arrest [5]. Reduced leaf area, necrosis, and abscission are visibly damaging effects of salinity stress [6]. Besides this, salinity stress drastically affects the uptake of key mineral ions including N, S, K, P, and Ca, thereby triggering hindrances in normal growth and development by impeding growth promotion [5,7]. Like other stress factors, salinity also triggers a considerable increase in reactive oxygen species (ROS) [8].

To counteract the damaging effects of salinity stress, indigenously existing tolerance mechanisms are up-regulated. These include (a) the antioxidant system to neutralize the excess accumulated ROS; (b) osmolyte accumulation for maintenance of tissue water potential, and (c) up-regulation of the expression of genes coding for crucial regulatory proteins controlling an array of physiological and biochemical pathways including ion uptake and salt exclusion [7,9,10]. The plant antioxidant system with enzymatic and non-enzymatic mechanisms protects the structural and functional stability of macromolecules such as proteins, lipids, and nucleic acids by preventing their oxidation, while osmolytes and secondary metabolites assist in ROS scavenging besides their emerging roles in stress signaling [11]. Optimal ROS and compatible osmolytes mediate nutrient and metabolite signaling, leading to the activation of specific transduction pathways for triggering modulation of gene expression and proteomic patterns [12]. It is known that salinity tolerance is a complex trait wherein several physiological, biochemical and molecular networks have been found to exert a well-coordinated role [13,14]. Up-regulation of stress tolerance mechanisms, including the antioxidant system, accumulation of osmolytes and secondary metabolites, salt exclusion, and expression of key stress-responsive genes, has been reported to contribute to growth and metabolism protection in several plants [5,7,10,15]. Nonetheless, several approaches have been proposed and adapted to improve plants’ naturally occurring stress tolerance mechanisms [16,17].

Myo-inositol (MYO) is a vital physiological metabolite common in all eukaryotic cells [18,19]. MYO forms the structural basis of many lipid signaling agents that regulate complex cellular signaling pathways, including responses to stress, biosynthesis of the cell wall and ascorbic acid, and auxin perception [20,21,22]. The synthesis of MYO is genetically regulated, and it has been reported that mutants exhibiting reduced MYO synthesis are much more sensitive to stresses [23] and show spontaneous cell death [24]. Recently, Hu et al. [25] reported that reduced synthesis of MYO causes cell death in leaves and roots. MYO regulates the production of stress molecules, cell to cell communication, phytohormone storage and transport, and P storage in seeds [18,22].

Quinoa (*Chenopodium quinoa* L.) is grown for its edible seeds and is commonly used therapeutically [26]. With visual signs of environmental changes and a growing human population expected in the upcoming years, quinoa could be a promising crop that helps solve some agricultural issues even while supplying secure and sustainable agricultural output [27]. Though quinoa is a facultative halophytic plant species, its growth and productivity can still be affected by excess salinity [28]. In terms of exposure to salinity seedlings, quinoa plants seem to be more sensitive to salt than mature plants [29]. However, the influence of MYO on the growth and photosynthetic regulation of salinity-stressed quinoa plants has not been studied to date. In the present study, we hypothesized that the application of MYO could regulate antioxidant and osmolyte metabolism and gene expression for improved salt stress tolerance in quinoa.

## 2. Materials and Methods

### 2.1. Experimental Setup and Treatment

Seeds of quinoa (*Chenopodium quinoa* L. var. Giza1) were obtained from Agricultural Research Centre Giza, Egypt. Seeds were immersed in 70% ethanol for 10 s, then surface sterilized with 2% NaOCl for 5 min, and thoroughly rinsed with double distilled water (DDW) three times. About 10 sterilized seeds were soaked in Petri dishes lined with filter paper moistened with 20 mL of Hoagland solution for 5 days in a germination incubator (PH070A) at 19 °C. After that, healthy seedlings were transferred into pots (15 cm diameter) filled with sterilized soil (clay and sand in 3:1 ratio), and an equal quantity of compost was added to each pot. Pots were irrigated with 100 mL Hoagland solution every alternate day for another 7 days. Then, seedlings per pot were thinned to one, and pots were divided into two groups. One group was foliarly treated with 10 mM MYO (Sigma-Aldrich; 50 mL per pot), which continued for 7 days, while the second group was not treated with MYO. Both MYO treated and untreated seedlings were treated with different concentrations of NaCl, i.e., 300, 450, and 600 mM, to induce salinity stress. Salinity treatment continued for another 2 weeks, and NaCl was given in the form of a modified nutrient solution, while unstressed pots received nutrient solution only. Pots irrigated with only Hoagland solution served as control. The pots were arranged in a completely randomized block design with four replicates for each treatment in a greenhouse having average relative humidity of 70–75%, photosynthetically active radiation of 750 ± 20 µmol m^−2^ s^−1^ day/night, temperatures of 25/18 °C and photoperiod of 12 h light/6 h dark. After 2 weeks of salinity stress (33-days after exposure to salinity), plant samples were harvested, and biochemical and physiological assays were performed.

### 2.2. Growth Measurements

Plant height (PH) was measured using a scale. Fresh weight of shoot and root was taken after harvesting, while dry weights were recorded after oven-drying the samples at 70 °C for 24 h. The number of surviving leaves per plant and leaf area (LA) were determined at the final harvesting time.

### 2.3. Measurement of Photosynthetic Pigments, Gas Exchange Parameters, and PSII Activity

The chlorophyll content was determined by the method of Lichtenthaler and Wellburn [30]. For photosynthetic measurement rate (Pn), stomatal conductance (gs), and transpiration rate (E), a portable infrared gas analyzer system (TPS-2, Amesbury, MA, USA) was used. The maximum quantum efficiency of PSII photochemistry (Fv/F_m_) was determined using a modulated chlorophyll fluorometer (PAM 2500; Walz, Germany).

### 2.4. Estimation of Stress Biomarkers

Lipid peroxidation was measured employing the method of Heath and Packer [31]. The superoxide anion (O_2_^−^) content was measured according to the method described by Elstner and Heupel [32]. Hydrogen peroxide (H_2_O_2_) content was determined by following the method of Velikova et al. [33]. Electrolyte leakage (EL) was measured in 0.2 g leaf segments (0.5 cm) according to Blum and Ebercon [34]. For measuring membrane stability index (MSI), the method described by Sairam [35] was followed. Briefly, 0.2 g leaf was placed in 10mL distilled water. One sample was heated at 40 °C for 30 min, and solution electrical conductivity (EC1) was recorded, while another sample was heated for 10 min at 100°, and EC2 was recorded. MSI was calculated using the following equation.

MSI (%) = {1 − (EC1/EC2)} × 100

### 2.5. Estimation of Abscisic Acid

The method by Siciliano et al. was used to determine the abscisic acid (ABA) concentration [36]. Briefly, 500 mg leaf tissue material was extracted (80% methanol containing 2% glacial acetic acid). After centrifugation at 13,000× *g* for 5 min at 4 °C, the supernatant was filtered through Whatman filter paper No. 1 and analyzed by HPLC. An aliquot of approx. 20 μL was injected into an ACE Ultra Core 2.5 Super C18 column at a flow rate of 0.5 mL min^−1^.

### 2.6. Estimation of Osmolytes

Proline content was estimated following Bates et al. [37]. For estimating the glycine betaine (GB) content, the method of Grieve and Grattan was followed [38]. Total soluble protein content was determined by following the method of Bradford [39] using bovine serum albumin as standard. Total soluble sugar content was estimated according to the modified method of Irigoyen et al. [40]. The method of Moore and Stein was used for the estimation of free amino acids [41].

### 2.7. Measurements of RWC and LWP

Estimation of the relative water content (RWC) was carried out following the protocol of Dionisio-Sese and Tobita [42].

RWC _(%)_ = [(FW − DW)/(TW − DW)] × 100

where FW is fresh mass, TW is turgid weight and DW is dry weight.

For leaf water potential (LWP), 10 sunlight-exposed mature leaves with full biological activity (maximum leaf area) were used. Measurement of leaf water potential was conducted using a psychrometer between 09:00 and 11:00 h.

### 2.8. Assay of Antioxidant Enzymes

To measure the activity of superoxide dismutase (SOD; EC 1.15.1.1), the method of Misra and Fridovichwas was employed [43]. Catalase activity (CAT; EC 1.11.1.6) was determined following Aebi [44]. To measure the activity of ascorbate peroxidase (APX, EC 1.11.1.11), a decline in absorbance was monitored at 290 nm for 3 min, according to Nakano and Asada [45]. The activity of glutathione reductase (GR; EC 1.6.4.2) was assayed by following the method of Smith et al. [46]. The activity of glutathione S-transferase (GST; EC: 2.5.1.18) was assayed employing the protocol of Habig et al. [47]. Glutathione peroxidase (GPX; EC 1.11.1.9, GPX) activity was determined according to Hossain et al. [48].

### 2.9. Estimation of Non-Enzymatic Antioxidants

The content of ascorbic acid (AsA) was determined according to Jagota and Dani [49]. The content of oxidized glutathione (GSSG) and reduced glutathione (GSH) in leaf samples was determined based on the enzymatic recycling described by Anderson [50].

### 2.10. RNA Extraction and Quantitative Real-Time PCR (qRT-PCR)

Total RNA from quinoa was extracted using a plant-RNA kit according to the manufacturer’s protocol. After quantifying the RNA purity, samples were treated with RNAase-free DNAase and reverse transcription was applied for cDNA synthesis using “Promega Germany kit”. The thermal cycler was programmed at 42 °C for 1 h and 72 °C for 20 min. After that, quantitative real-time PCR was carried out in a 20 µL reaction mixture using a real-time analysis (Rotor-Gene 6000, Qiagen, Hilden, Germany) system. The primer sequences used are given in Table 1, and the β-Actin gene was used as an internal control. The relative gene expression was determined using the 2-ΔΔCt method of Livak and Schmittgen [51].

### 2.11. Statistical Analysis

Data were analyzed statistically using analysis of variance (ANOVA) by SPSS 17.0 for Windows and presented as mean ± SE (*n* = 4). The least significant difference (LSD) was calculated at *p* ≤ 0.05.

## 3. Results

**Figure 1:**
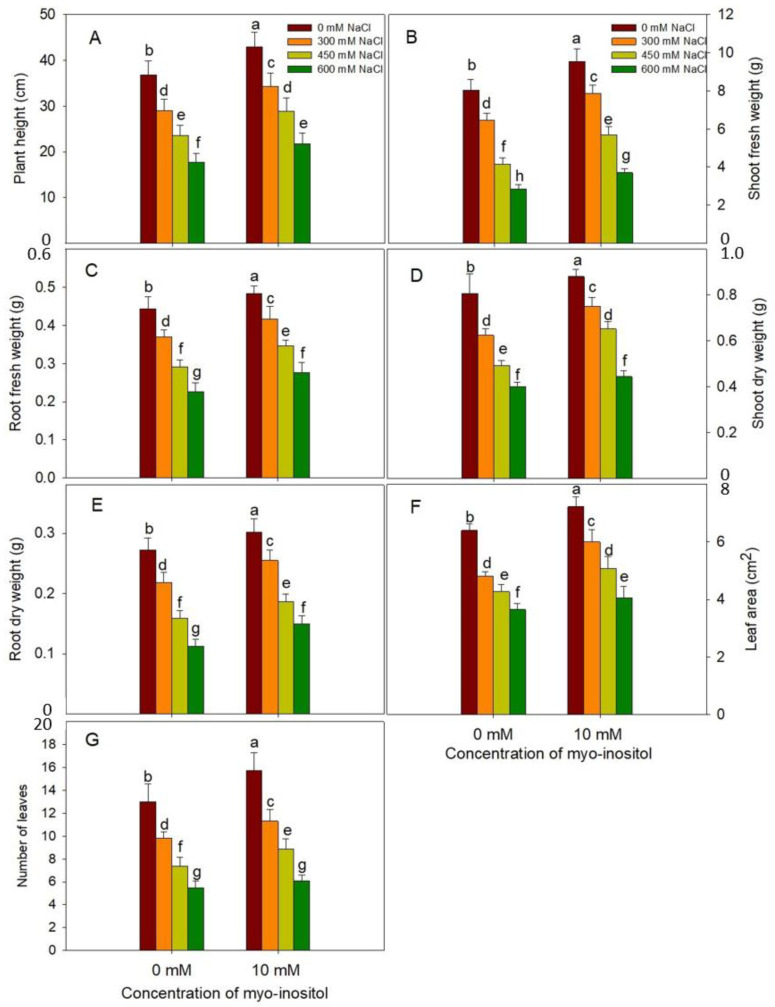
Effect of different salinity (300, 450, and 600 mM NaCl) concentrations with and without exogenous application of myo-inositol (10 mM) on growth parameters in Quinoa (*Chenopodium quinoa* L. var. Giza1). Data were expressed as (**A**) plant height (cm); (**B**) fresh shoot weight; (**C**) fresh root weight; (**D**) dry shoot weight; (**E**) dry root weight; (**F**) leaf area (cm^2^) and (**G**) leaf number. Values are mean (±SE) of four replicates, and different letters represent significant differences at *p* ≤ 0.05.

Current work shows the effect of salinity stress and exogenous application of MYO on growth parameters such as plant height, fresh and dry weight of shoot and root, LA, and leaf number (Figure 1 and Supplementary Materials Figure S1). Salinity treatments reduced all the growth parameters significantly with maximal decline at 600 mM NaCl. Relative to control, at 600 mM NaCl, the percent decline in PH was 51.84%; fresh shoot weight, 49.01%; dry shoot weight, 50.26%; fresh root weight, 46.77%; dry root weight, 65.17%; LA, 42.97%; and leaf number, 54.47%, respectively. However, application of 10 mM MYO resulted in an increase of 16.77% in PH; 17.07% and 9.26% in fresh and dry weight of shoot, respectively; 10.13% and 8.45% in fresh and dry weight of root, respectively; 12.85% in LA; and 30.43% in leaf number over the control counterparts at 600 mM NaCl. Our results show that the exogenous application of MYO alleviates the adverse effects of salinity (Figure 1).

**Figure 2:**
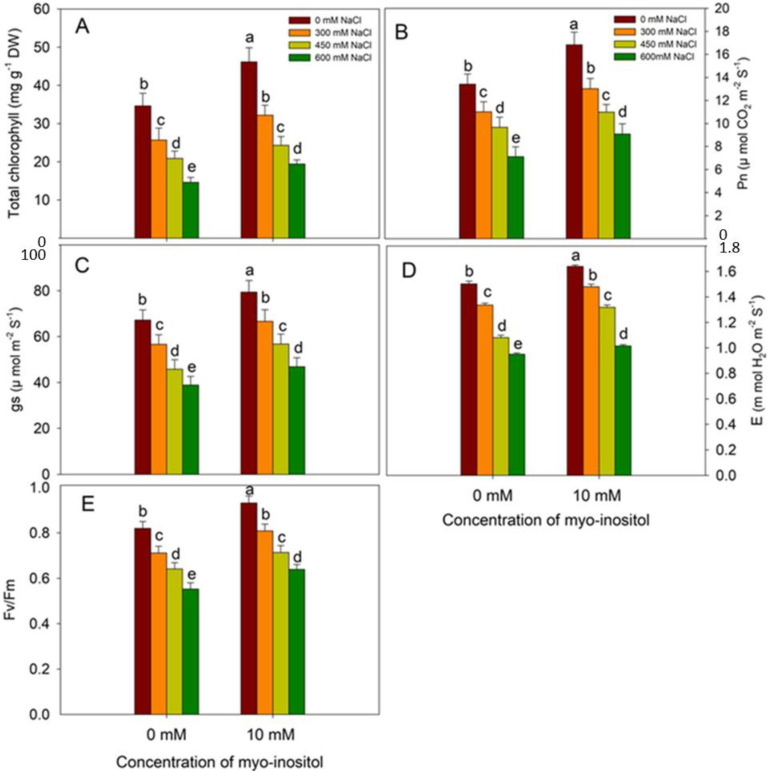
Effect of different salinity (300, 450, and 600 mM NaCl) concentrations with and without exogenous application of myo-inositol (10 mM) on changes in photosynthetic attributes and gas exchange in Quinoa (*Chenopodium quinoa* L. var. Giza1). Data expressed as (**A**) total chlorophyll content (Chl); (**B**) net photosynthetic rate (*Pn*); (**C**) stomatal conductance (gs); (**D**) transpiration rate, and (**E**) photosynthetic efficiency (*Fv/Fm*). Values are mean (±SE) of four replicates, and different letters represent significant differences at *p* ≤ 0.05.

Besides this, salinity-stressed quinoa exhibited a decrease in chlorophyll content, *Pn*, gs, E, and Fv/Fm compared to the control and MYO-treated seedlings (Figure 2). However, exogenous application of MYO caused an enhancement of 33.38% in total chlorophyll, 25.50% in *Pn*, 15.34% in gs, 9.11% in E, and 12.01% in Fv/Fm as compared to control. Application of MYO to salinity-stressed counterparts resulted in considerable amelioration of the decline at all concentrations of NaCl, thereby depicting substantial enhancement over the respective saline-stressed counterparts (Figure 2).

**Figure 3:**
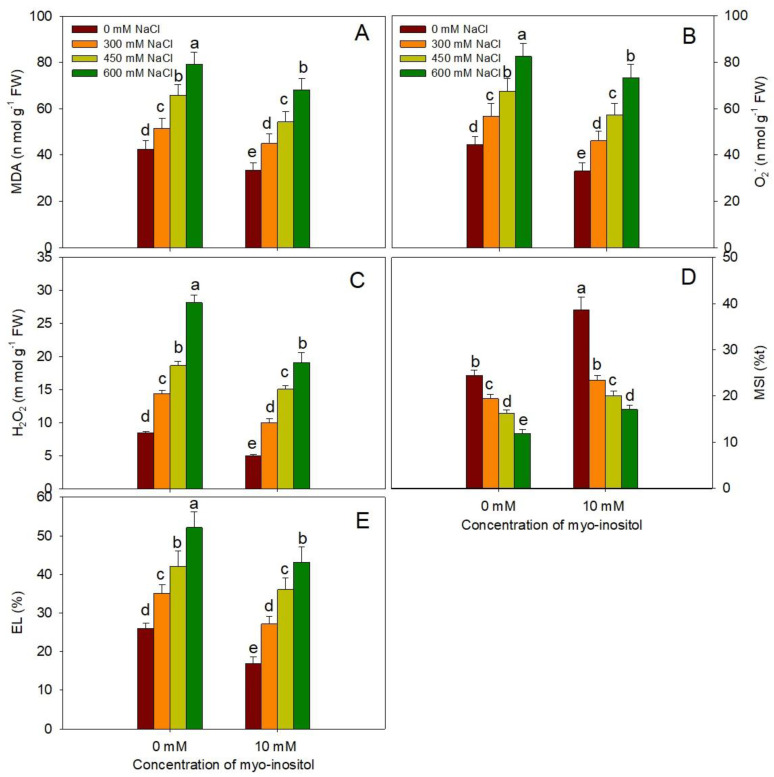
Effect of different salinity (300, 450, and 600 mM NaCl) concentrations with and without exogenous application of myo-inositol (10 mM) on changes in oxidative damage attributes in Quinoa (*Chenopodium quinoa* L. var. Giza1). Data expressed as (**A**) malondialdehyde content (MDA); (**B**) superoxide ion (O^2−^); (**C**) hydrogen peroxide (H_2_O_2_); (**D**) membrane stability index (MSI), and (**E**) electrolyte leakage (EL). Values are mean (±SE) of four replicates, and different letters represent significant differences at *p* ≤ 0.05.

Exogenous application of myo-inositol also helped to protect the plant from oxidative damage of abiotic stress. Relative to control, contents of O_2_^−^, H_2_O_2_, MDA, and EL maximally increased by 85.39%, 231.75%, 85.97%, and 100.10%, respectively, at 600 mM NaCl treatment. However, the application of MYO resulted in a decrease of 25.73%, 40.73%, 21.59%, and 35.07% in O_2_^−^, H_2_O_2_, MDA, and EL, respectively, while MSI increased by 56.32% over control in plants subjected to salinity stress. The results show that MYO application to NaCl-stressed seedlings mitigated the oxidative damage by decreased O_2_^−^ and H_2_O_2,_ thereby reducing MDA and EL with concomitant enhancement in MSI at all NaCl concentrations (Figure 3).

**Figure 4:**
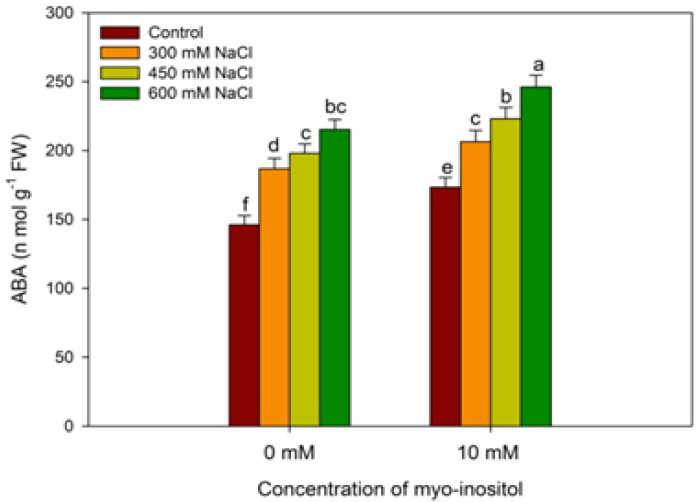
Effect of different salinity (300, 450, and 600 mM NaCl) concentrations with and without exogenous application of myo-inositol (10 mM) on changes in abscisic acid (ABA) in Quinoa (*Chenopodium quinoa* L. var. Giza-1). Values are mean (±SE) of four replicates, and different letters represent significant differences at *p* ≤ 0.05.

Plants analyzed for ABA content showed an increase with increasing salt concentration. Relative to control, ABA increased by 27.80%, 35.61%, and 47.46% at 300, 450, and 600 mM NaCl, respectively, and a further increase of 10.55%, 12.62%, and 14.25% was imparted due to the application of MYO over the respective NaCl-stressed counterparts. The MYO application resulted in an increase of 18.69% in ABA over control (Figure 4).

**Figure 5:**
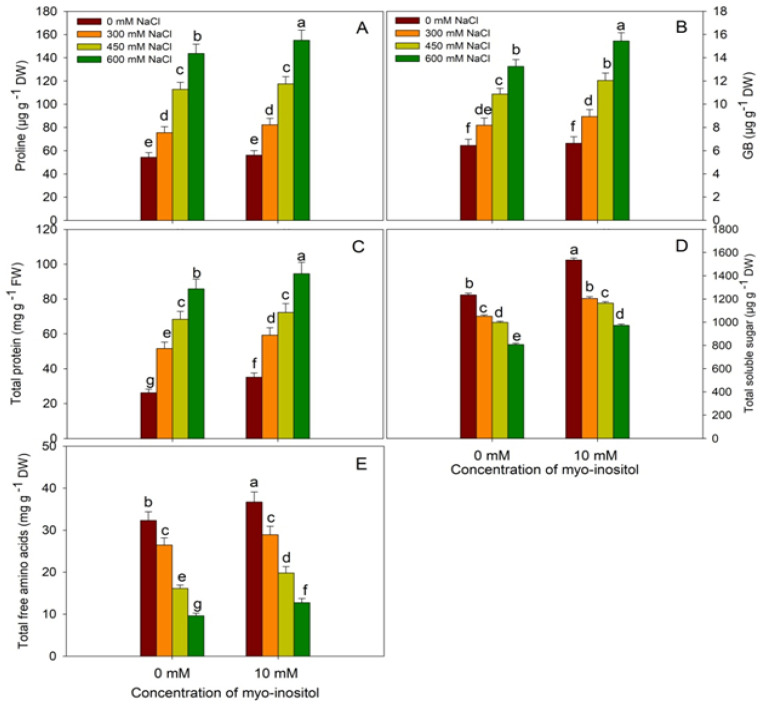
Effect of different salinity (300, 450, and 600 mM NaCl) concentrations with and without exogenous application of myo-inositol (10 mM) on changes in osmolyte concentration in Quinoa (*Chenopodium quinoa* L. var. Giza1). Data expressed as (**A**) total proline content; (**B**) glycine betaine (GB); (**C**) total protein; (**D**) total soluble sugars (TSS), and (**E**) total free amino acids. Values are mean (±SE) of four replicates, and different letters represent significant differences at *p* ≤ 0.05.

Salinity stress in quinoa resulted in the accumulation of proline, GB, and total proteins; however, soluble sugars and free amino acids exhibited declines (Figure 5). Relative to control, proline, GB, and total protein increased by 164.59%, 105.74% and 227.59%, respectively, under 600 mM NaCl stress. Application of MYO caused further enhancement in their content at all NaCl concentrations, attaining maximal increases of 185.63%, 139.75%, and 260.91%, respectively, at 600 mM NaCl plus MYO treated seedlings over control. Under normal conditions, proline increased by 3.27%, GB by 2.95%, total protein by 33.96%, soluble sugars by 24.29%, and free amino acid by 13.65% due to MYO application over control. However, the contents of soluble sugars and free amino acids decreased with increasing NaCl concentrations, attaining maximal declines of 34.57% and 70.24% at 600 mM NaCl and did not exhibit any significant difference with exogenous application of myo-inositol (Figure 5).

**Figure 6:**
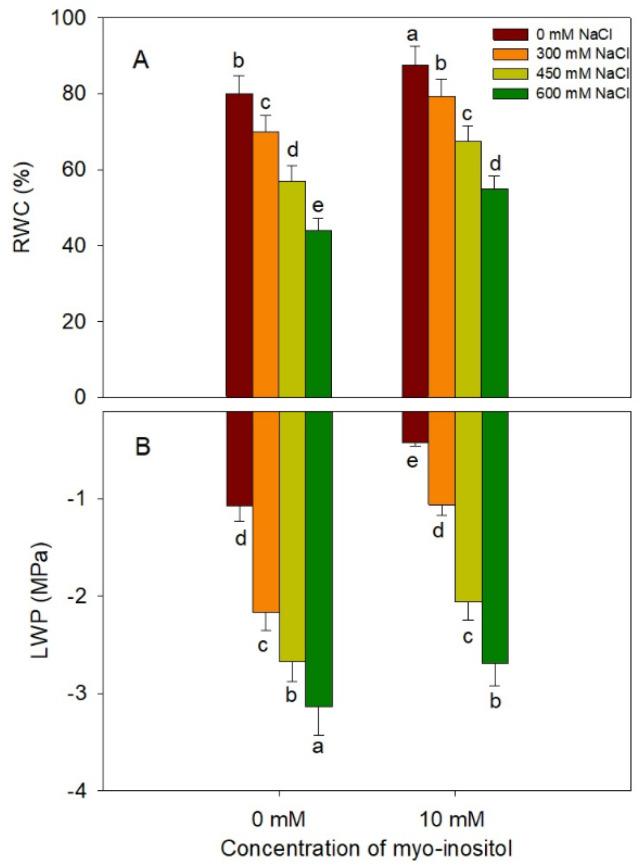
Effect of different salinity (300, 450, and 600 mM NaCl) concentrations with and without exogenous application of myo-inositol (10 mM) on changes in water relations in Quinoa (*Chenopodium quinoa* L. var. Giza1). Data expressed as (**A**) relative water content (RWC) (%) and (**B**) leaf water potential (LWP; Mpa). Values are mean (±SE) of four replicates, and different letters represent significant differences at *p* ≤ 0.05.

Salinity stress has a negative impact on RWC and LWP in plants under saline conditions. A similar pattern of significant decline was observed in our experiment due to salinity stress, with a maximal decline at 600 mM NaCl; however, application of MYO ameliorated the decline to a considerable extent under all salinity levels (Figure 6). MYO (10 mM) application increased RWC and LWP and mitigated the decline significantly at all salinity concentrations (Figure 6).

**Figure 7:**
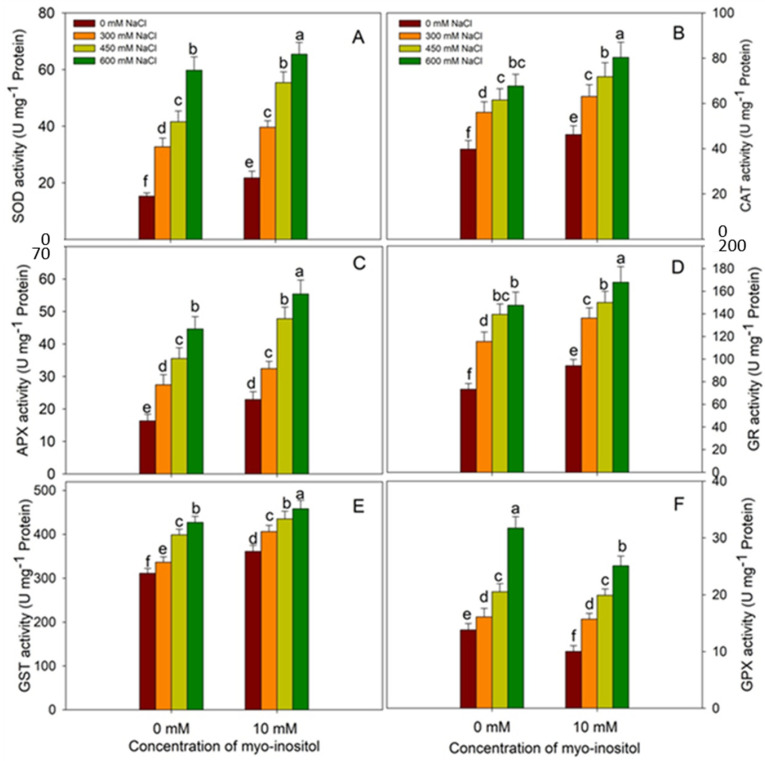
Effect of different salinity (300, 450, and 600 mM NaCl) concentrations with and without exogenous application of myo-inositol (10 mM) on changes in enzymatic antioxidant status and activities in Quinoa (*Chenopodium quinoa* L. var. Giza1). Data expressed as (**A**) superoxide dismutase (SOD, EC 1.15.1.1); (**B**) catalase (CAT, EC1.11.1.6); (**C**) ascorbate peroxidase (APX, EC 1.11.1.11); (**D**) glutathione reductase (GR); (**E**) glutathione-S-transferase (GST; EC 2.5.1.18), and (**F**) guaiacol peroxidase (EC 1.11.1.9, GPX). Values are mean (±SE) of four replicates, and different letters represent significant differences at *p* ≤ 0.05.

The application of MYO resulted in significant up-regulation of the activity of antioxidant enzymes. Relative to control, the activity of SOD increased by 41.73%, CAT by 16.06%, APX by 40.46%, GR by 28.38%, and GST by 16.05% in MYO-treated seedlings (Figure 7). Salinity stress increased the activity of antioxidants in a concentration-dependent manner with maximal increase of 290.01%, 69.97%, 173.57%, 101.14%, 37.15%, and 129.71% in SOD, CAT, APX, GR, GST, and GPX, respectively, at 600mM NaCl over control. Application of MYO to salinity-stressed plants caused a further increase in their activities over their respective salinity-stressed counterparts. Relative to control, maximal enhancement in the activity of antioxidant enzymes was 327.30% for SOD, 101.65% for CAT, 239.60% for APX, 128.80% for GR, and 47.14% for GST in plants treated with 600 mM NaCl plus MYO (Figure 7).

**Figure 8:**
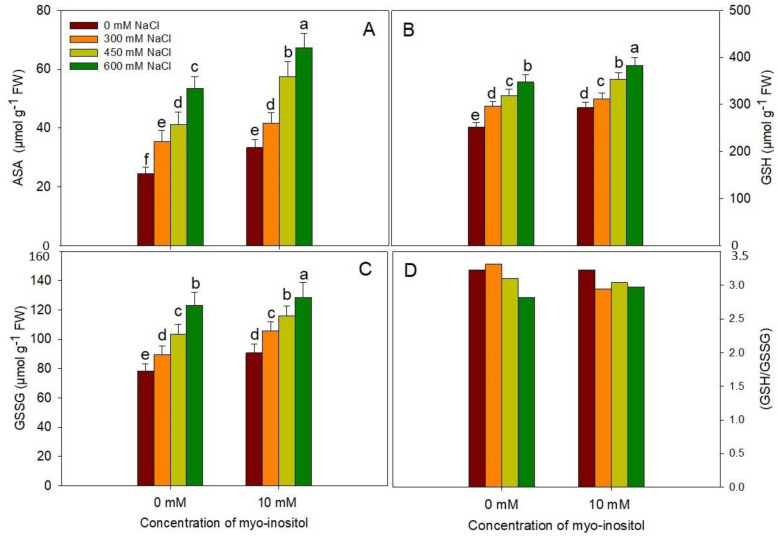
Effect of different salinity (300, 450, and 600 mM NaCl) concentrations with and without exogenous application of myo-inositol (10 mM) on changes in non-enzymatic antioxidant status and activities in Quinoa (*Chenopodium quinoa* L. var. Giza1). Data were expressed as (**A**) total ascorbate (AsA); (**B**) total glutathione (GSH); and (**C**) total oxidized glutathione (GSSG) and (**D**) GSH/GSSG. Values are mean (±SE) of four replicates, and different letters represent significant differences at *p* ≤ 0.05.

The contents of AsA, GSH, and GSSG also increased with salinity stress and attained a maximal increase of 119.26%, 37.77%, and 57.11%, respectively, at 600 mM NaCl treatment. Application of MYO imparted an increase under normal conditions as well as under salinity stress (Figure 8). However, exogenous application of myo-inositol did not show any significant difference in GSH and GSSG (GSH/GSSG) ratios in all treated plants (Figure 8D).

**Figure 9:**
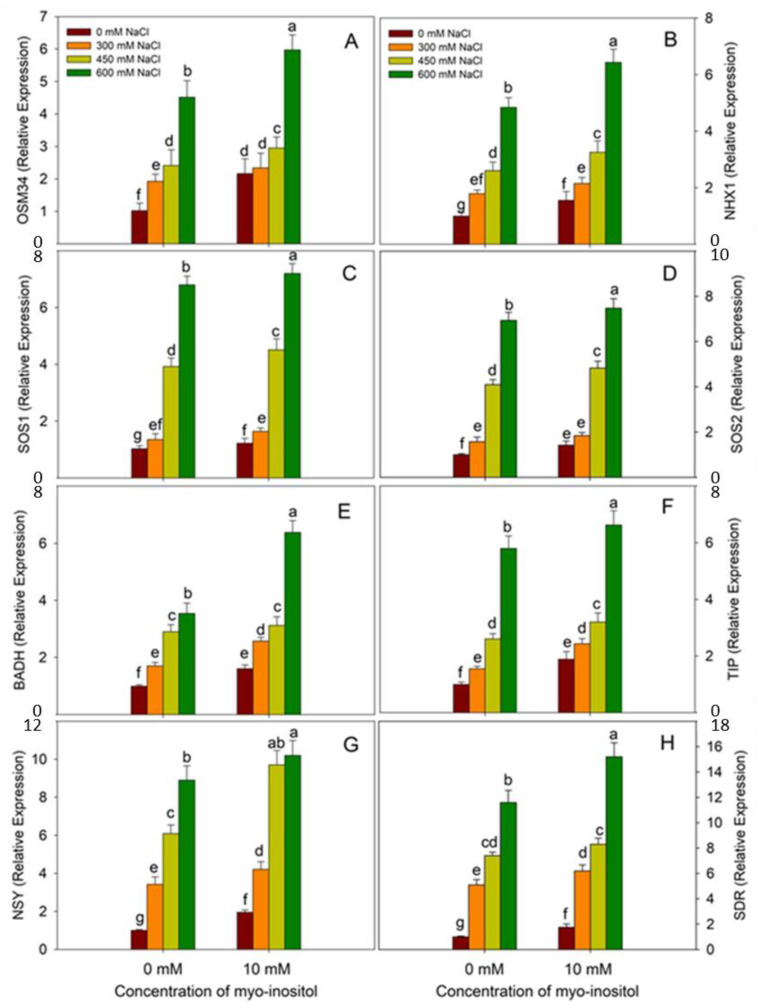
Effect of different salinity (300, 450, and 600 mM NaCl) concentrations with and without exogenous application of myo-inositol (10 mM) on changes in relative gene expression of salinity-stressed proteins and genes involved in ABA and glycine betaine biosynthesis in Quinoa (*Chenopodium quinoa* L. var. Giza1). Data expressed as (**A**) osmotin-like protein (OSM34); (**B**) sodium/proton exchanger (Na/H^+^) (NHX1); (**C**) salt overly sensitive 1A (SOS1A); (**D**) salt overly sensitive 1B (SOS1B); (**E**) betaine aldehyde dehydrogenase (cqBADH); (**F**) tonoplast intrinsic protein 2 (TIP2); (**G**) neoxanthin synthase (NSY); and (**H**) short chain-dehydrogenase/reductases (SDR). Values are mean (±SE) of four replicates, and different letters represent significant differences at *p* ≤ 0.05.

**Figure 10:**
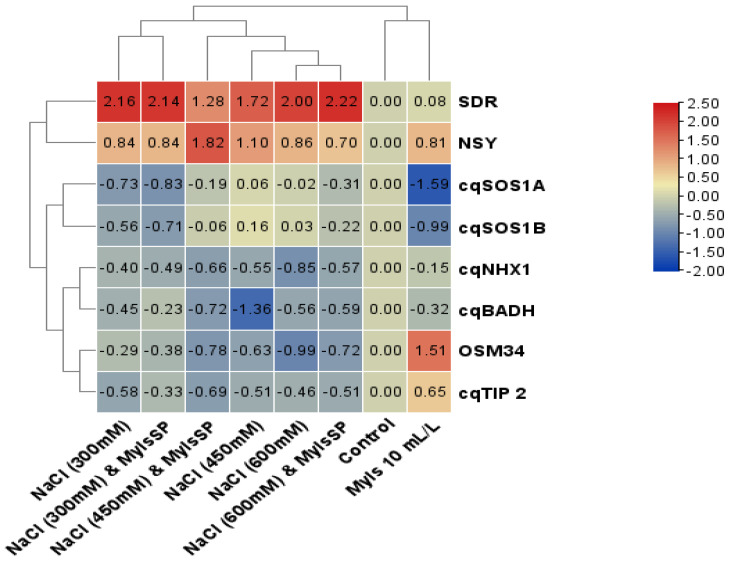
Transcriptomic patterning and gene expression of salinity-stressed proteins and genes involved in ABA and glycine betaine biosynthesis in Quinoa (*Chenopodium quinoa* L. var. Giza1). Genes involved are osmotin-like protein (OSM34); sodium/proton exchanger (Na/H^+^) (NHX1); salt overly sensitive 1A (SOS1A); salt overly sensitive 1B (SOS1B); betaine aldehyde dehydrogenase (cqBADH); tonoplast intrinsic protein 2 (TIP2); neoxanthin synthase (NSY); and (H) short chain-dehydrogenase/reductases (SDR).

The expression analysis of genes including *OSM34*, *NHX1*, *SOS1A*, *SOS1B*, *BADH*, *TIP2*, *NSY,* and *SDR* revealed that application of myo-inositol enhanced their expression by 2.11, 1.56, 1.19, 1.43, 1.61, 1.89, 1.95, and 1.75 fold, respectively, over the control (Figure 9). It was observed that salinity stress increased the expression of these genes at all concentrations. Relative to control, maximal increases of 4.42 fold for *OSM34*, 4.87 fold for *NHX1*, 6.62 fold for *SOS1A*, 6.97 fold for *SOS1B*, 3.57 fold for *BADH*, 5.85 fold for *TIP2*, 8.92 fold for *NSY,* and 11.67 fold for *SDR* at 600 mM NaCl were observed. MYO application to NaCl-treated seedlings further increased their expression over the respective NaCl-stressed counterparts, attaining a maximal increase in 600 mM NaCl plus MYO treated seedlings (Figure 10).

## 4. Discussion

Salinity stress is causing serious damage to global food security, and various key strategies have been devised to strengthen the indigenous tolerance potential of plants. Modulation in the tolerance mechanisms through the application of protectants has been considered an effective strategy to better assist plants in withstanding stress conditions. The present study revealed the beneficial influence of an exogenously applied metabolite, MYO, in quinoa under salinity stress. Salinity stress reduces the growth of cells, cell proliferation by declining cell cycle genes [2,52], mitotic index, and relative cell division rate [53]. Salinity stress reduces growth by inducing osmotic and ionic stress, resulting in hampered cellular functioning, thereby restricting plant growth [3]. Earlier salinity-mediated declines in growth and, fresh and dry weight have been reported [1,7]. Recently, Dell Aversana et al. [54] also demonstrated a significant decline in growth and weight production in salinity-stressed barley genotypes concomitant with a reduction in water potential. Salinity stress-mediated excess accumulation of toxic ions such as Na^+^ limits plant growth by restricting enzyme functioning, water balance, and photosynthesis [55]. These findings agree with our findings that plants, when subjected to saline conditions, exhibited reduced morphological parameters compared to control. However, the application of MYO resulted in increased growth in terms of PH and fresh and dry weight of root and shoot. Hu et al. also demonstrated significant alleviation of salinity-mediated decline in growth in *Malushu pehensis* with MYO application [56]. Increased salinity tolerance in MYO-treated plants is attributed to the over-expression of key transport and signaling genes such as *NHX*, *HKT1*, *SOS1,* and *SOS2,* leading to the maintenance of ion homeostasis and preventing the toxic effects of salt ions [56]. Our results agree with this statement, as the application of MYO resulted in the up-regulation of *NHX1*, *SOS1A,* and *SOS1B* genes under normal conditions and salinity stress. *NHX* acts as Na/H and K/H antiporters, and is essential in maintaining cellular ion and pH homeostasis, thereby preventing ion toxicity under salinity stress and contributing to K ion concentration maintenance [57]. Recently, Sun et al. demonstrated that increased salinity tolerance in soybean over-expressing *NHX* gene reflected reduced oxidative damage and increased *SOS1*, *SKOR,* and *HKT* [58]. The regulated expression of *NHX* and *SOS* maintains the ratio of Na/K, thereby significantly affecting the growth under saline conditions [59,60]. Improving Na^+^ exclusion leads to maintaining ion homeostasis in roots, thus ensuring relatively lower concentrations of toxic ions within shoot [61]. Increased expression of *SOS* genes under salinity has also been reported by Sathee et al. [62] in wheat and Ma et al. [63] in *Vitis vinifera*. Maintenance of lower cellular concentrations of toxic ions protects major cellular pathways, including photosynthesis, by maintaining their structural and functional integrity [56]. In addition, exogenous application of MYO improved the expression of the *OSM34* (osmotin) gene under normal and salinity stress conditions, which eventually enhanced the alleviation of stress. Osmotin is one of the essential stress-responsive genes known to prevent cell damage and growth by reducing ROS accumulation, lipid peroxidation, and programmed cell death while increasing proline accumulation [64]. Over-expression of osmotin has been reported to confer salinity tolerance to tomatoes, resulting in enhanced growth by maintaining the tissue water content [65]. In the present study, MYO-induced up-regulation of *OSM34* may have contributed to improved growth under salinity stress by maintaining RWC. However, reports discussing the influence of MYO on *OSM34* expression are not available. Tonoplast intrinsic proteins (*TIP*) are generally targeted to the vacuolar membrane and act as water channels for facilitating water transport across subcellular compartments. *TIP* isoforms are involved in the translocation of H_2_O_2_, glycerol, and urea, besides improving the permeability of vacuolar membranes to ammonia [66,67]. Increased TIP expression may also enhance the hydraulic conductivity for maintaining greater water uptake, thereby alleviating salinity stress-induced osmotic effects [68]. Short-chain dehydrogenase/oxidoreductase (*SDR*) plays an essential role in the salinity stress tolerance of plants [69]. In the present study, MYO up-regulated its expression, which could have contributed to the regulation of ABA biosynthesis [70].

Moreover, the expression pattern of neoxanthin synthase (*NSY*) increased due to the application of MYO under normal and salinity stress conditions, thereby contributing to an increase in the synthesis of neoxanthin, which acts as a precursor for ABA biosynthesis. This increased *NSY* expression could have regulated the ABA-mediated signaling events [71,72]. Moreover, it can also contribute to photo-protection [73]. An increase in ABA due to salinity was reported earlier by Kwon et al. [74].

Our results also show that chlorophyll content exhibited a significant decline in salinity-stressed seedlings; however, the application of MYO resulted in mitigating the reduction to some extent. In the same way, *Pn*, gs, E, and PSII activity was enhanced due to MYO exogenous application and amendment of the saline regime. Similarly to our results, a decline in chlorophyll content, *Pn*, gs, E, and PSII activity has been reported in different plants [7,75,76,77]. In salt-stressed *Dianthus caryophyllus*, Kwon et al. [74] demonstrated a decline in *Pn*, gs, E, intercellular CO_2_, and stomata cavity in the lower and upper epidermis, with a significant decrease in their size and density. The decline in photosynthetic performance under salinity stress was attributed to altered K/Na [78]. The reduction in photosynthesis due to salinity stress could be due to the excess ROS accumulation [79] and lowered tissue water potential [80]. It has been concluded that salinity stress decreases chlorophyll biosynthesis by affecting the activity of key biosynthetic enzymes [81]. Reports discussing the role of MYO in chlorophyll and photosynthetic regulation are rare. Sarropoulou et al. reported an increase in chlorophyll synthesis in sweet cherries due to supplementation with MYO in the tissue culture medium [82]. Pre-treatment with MYO in *Malushupehensis* alleviated the decline in chlorophyll and photosynthesis under salinity stress [56]. Increased chlorophyll and photosynthesis due to exogenous application of MYO can be attributed to the up-regulation of chlorophyll biosynthetic enzymes with a concomitant reduction in ROS accumulation and chlorophyll degradation.

In addition, the application of MYO ameliorated oxidative damage by reducing the accumulation of toxic ROS, including H_2_O_2_ and O_2_^·−^, resulting in reduced lipid peroxidation and EL with a concomitant increase in the MSI. In corroboration with our findings, earlier Elkelish et al. reported a significant increase in ROS accumulation, MDA, and EL under salinity stress in wheat [7]. Stresses result in loss of membrane functional and structural stability by triggering the peroxidation of membrane proteins and lipids, thereby decreasing the content of polyunsaturated fatty acids [83]. Stress-mediated alteration in membrane functioning is also due to the up-regulation of lipoxygenase activity [84]. Recently, Munawar et al. [85] observed oxidative damage to cotton due to salinity stress. Hu et al. demonstrated that apple plants deficient in MYO synthesis showed excessive accumulation of ROS, thereby exhibiting extensive programmed cell death [25]. In the present study, MYO-induced alleviation of oxidative damage in terms of reduced lipid peroxidation and EL concomitant with increased MSI could be attributed to reduced accumulation of H_2_O_2_ and O_2_^·−^. In addition, exogenous application of MYO may maintain the lowered activity of lipoxygenase, resulting in maintenance of sufficient concentrations of polyunsaturated fatty acids. In the present study, alleviation of salinity-induced oxidative stress due to exogenous application of MYO could be due to up-regulation of the antioxidant system in *Chenopodium quinoa*. Exposure of *Chenopodium quinoa* to salinity stress resulted in increased activity of antioxidant enzymes and the accumulation of non-enzymatic components; however, application of MYO further increased their activities. This strengthened the antioxidant system for better elimination of ROS, thereby mitigating oxidative effects on key macromolecular functions. Similarly to our findings, salinity stress-induced up-regulation of the antioxidant system has been reported [7,79,86]. Plants exhibiting higher antioxidant functioning show greater tolerance to stress [87], and up-regulation of the antioxidant system due to applied MYO may have protected *Chenopodium quinoa* by maintaining low ROS levels and redox homeostasis, thereby protecting growth and development. The O_2_^·−^ radical is neutralized by SOD, while H_2_O_2_ is eliminated by CAT, peroxidase, or the ascorbate-glutathione (AsA-GSH) cycle. The MYO application resulted in up-regulation of the CAT, GPX, and AsA-GSH cycle components, maintaining structural and functional integrity of the cell. Up-regulation of the AsA-GSH cycle protects growth and cellular functioning by (a) maintaining redox homeostasis and (b) electron transport [88]. Exogenous application of MYO to bentgrass has been reported to up-regulate antioxidant enzyme activity and gene expression, resulting in increased photosynthesis and water use efficiency under drought stress [89]. The up-regulation of the AsA-GSH cycle significantly improves cellular functioning and photosynthesis by quickly eliminating ROS, leading to redox homeostasis maintenance and other defense systems [90].

Increased AsA-GSH functioning in MYO-treated seedlings contributed to the maintenance of increased AsA and GSH content. AsA and GSH act as potent non-enzymatic antioxidants [91]. Under stress conditions, raised GSH protects membranes by maintaining the reduced state of both α-tocopherol and zeaxanthin, and also prevents the oxidative denaturation of proteins by protecting their thiol groups [92]. Redox state measured as GSH/GSSG decreased due to NaCl treatment, while MYO-treated plants exhibited a slightly increased GSH/GSSG ratio. However, these results were not so noticeable and therefore need further experimentation. Maintaining redox homeostasis may have significantly contributed to plant growth and development. GSH acts as a substrate for both GPX and GST. In the present study, MYO induced an increase in GSH, which may have contributed to increased GST activity concomitant with greater activity of AsA-GSH cycle enzymes. MYO has been reported to directly influence AsA synthesis, imparting stress tolerance in transgenic *Arabidopsis* seedlings exhibiting increased MYO synthesis [93].

Exogenous application of MYO resulted in increased accumulation of compatible osmolytes, including proline, GB, soluble sugars, and free amino acids, thereby contributing to tissue water potential maintenance, reducing deleterious salinity-induced ionic effects. Salinity stress-induced accumulation of osmolytes has been reported previously in several crop plants [94,95]. Increased accumulation of GB in transgenic *BADH* over-expressing wheat seedlings increases photosynthesis by protecting the thylakoid membranes [94]. In the present study, the application of MYO resulted in significant up-regulation of *BADH* gene expression, thereby contributing to the increased synthesis of GB. The modulations directly regulate the accumulation of osmolytes in the enzymes involved in their synthesis, and it has been reported that biosynthetic enzymes are up-regulated, while catabolic ones are down-regulated [95]. Under stressful conditions, osmolyte accumulation may contribute to osmoprotectants, ROS scavenging, protection of enzyme structures and functioning, stabilization of membranes, and maintenance of redox balance [96,97]. Sugars contribute to the up-regulation of growth-related genes with concomitant down-regulation of stress genes, thereby playing dual roles in the regulation of metabolism [98]. Maintaining a high sugar content protects photosystem structures and enhances photosynthetic performance by activation of sucrose synthetase and reduction of sucrose degradation [99,100]. These results indicate the beneficial impact of MYO application in improving tolerance to salinity stress in quinoa plants.

## 5. Conclusions

The present work on quinoa (*Chenopodium quinoa* L. var. Giza1) showed that salinity decreased most of the biochemical and morpho-physiological characteristics in a dose-dependent manner. The treatment of plants with MYO showed a significant increase in growth and photosynthetic traits and ameliorated salt-induced declines to a considerable extent. MYO augmented the ROS scavenging system via an elevated antioxidant defense system and osmolytes, which in turn improved growth. The expression analysis of *OSM34*, *NHX1*, *SOS1A*, *SOS1B*, *BADH*, *TIP2*, *NSY,* and *SDR* showed an increment under MYO application in plants under salt stress and non-stress conditions.

## Figures and Tables

**Table 1:** The sequences of the primers used in qRT-PCR.

Gene Name		Primer Sequence (5′–3′)
Osmotin-like protein (Osmotin-34)	F	GAACGGAGGGTGTCACAAAATC
	R	CGTAGTGGGTCCACAAGTTCCT
Tonoplast-localized Na+/H+ exchanger 1 (cq*NHX1*)	F	GCACTTCTGTTGCTGTGAGTTCCA
	R	TGTGCCCTGACCTCGTAAACTGAT
Salt overlay sensitive 1 (*SOS1A*)	F	CCTCATGATGCTTCCGACAA
	R	CCGAGTCAAGTGCTTCATCA
Salt overlay sensitive 1 (*SOS1B*)	F	ACCCTCATGATGCTTCTGATAC
	R	TGCTTCATCAACTGATTGCAT
Betaine aldehyde dehydrogenase (cq*BADH*)	F	GGTTACAGTCATTCAGACACCATCA
	R	AACAAAGGGAGCCAAGCAGTT
Tonoplast intrinsic protein 2 (*TIP2*)	F	AGTCCACCACCGATAAGAGGACCA
	R	CCACATCCATGCAAATATGGAAAGAGGA
Short-chain alcohol dehydrogenases/reductases (*SDR*)	F	CAATCTTGGCCAGCATCTCT
	R	CCAGCTAACCCAGCATTGTT
Neoxanthin synthase (*NSY*)	F	TTGTCTTGGACACCTGACACA
	R	CTCCAGTCCGTCATGGAAAA
β-Actin	F	GTGCCCATTTACGAAGGATA
	R	GAAGACTCCATGCCGATCAT

## Supplementary Materials

The following are available online at https://www.mdpi.com/article/10.3390/plants10112416/s1, Figure S1: Effect of different salinity (300, 450, and 600 mM NaCl) concentrations with and without exogenous application of MYO-inositol (10 mM) on growth parameters in Quinoa (*Chenopodium quinoa* L. var. Giza1).
