# Supplementary material for: Exogenous Myo-Inositol Alleviates Salt Stress by Enhancing Antioxidants and Membrane Stability via the Upregulation of Stress Responsive Genes in Chenopodium quinoa L
Source: Plants (Basel). 2021 Nov 9;10(11):2416. doi: 10.3390/plants10112416 (PMC8623490; doi:10.3390/plants10112416)

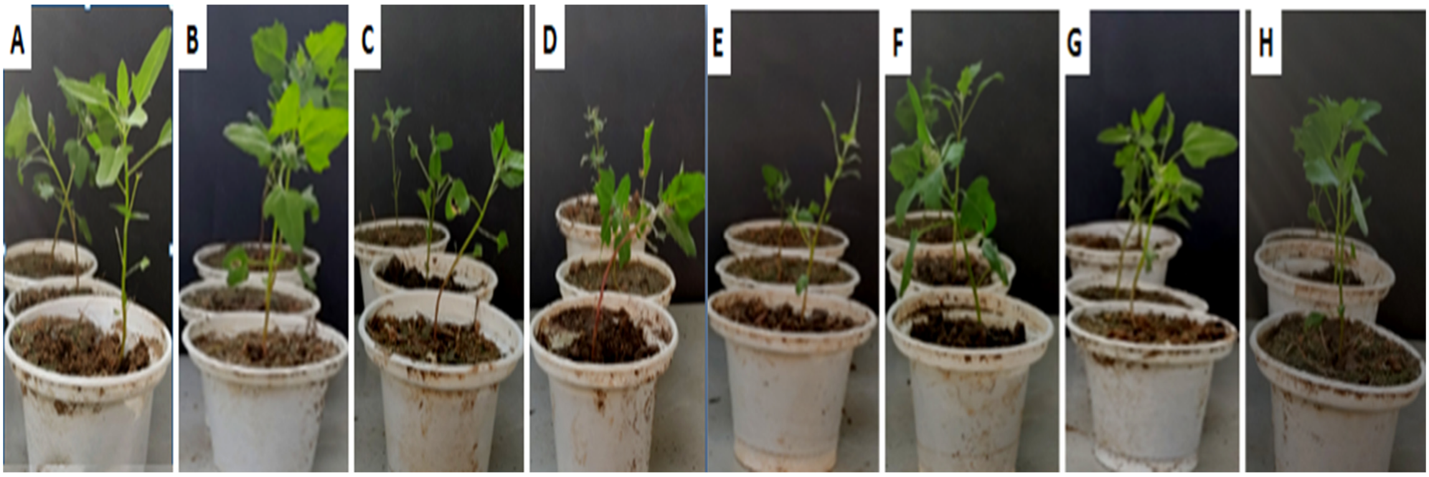

Supplementary Figure S1. Effect of different salinity (300, 450, and 600 mMNaCl) concentrations with and without exogenous application of MYO-inositol (10 mM) on growth parameters in Quinoa (*Chenopodium quinoa* L. var. Giza1). Figures shows effect of salinity and MYO application under control and saline regimes where (A) Control ; (B) Myoinositol 10mM; (C)300mM NaCl; (D) 450mM NaCl; (E) 600mM NaCl; (F)300mM NaCl+Myoinositol; (G)450mM NaCL+Myoinositol; (H) 600mM NaCl+Myoinositol.

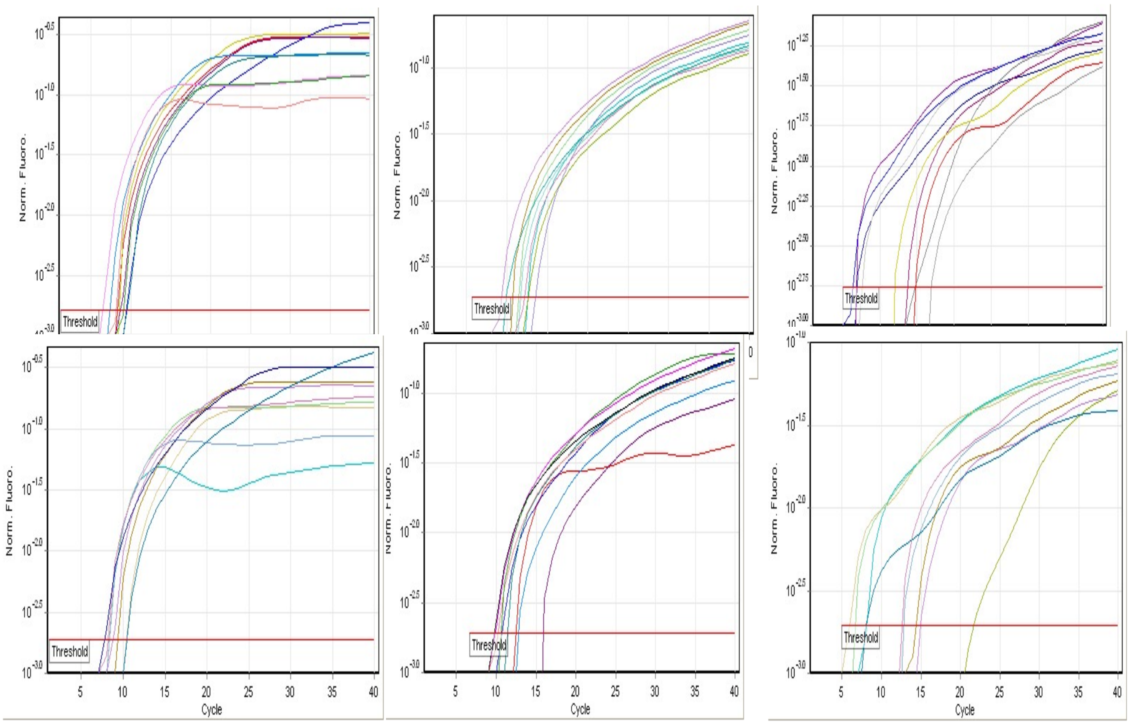

Supplement: Supplementary file 1 [file plants-10-02416-s001.zip › plants-1426622-supplementary.pdf]
